# Persistent Antiphospholipid Antibodies Are Not Associated With Worse Clinical Outcomes in a Prospective Cohort of Hospitalised Patients With SARS-CoV-2 Infection

**DOI:** 10.3389/fimmu.2022.911979

**Published:** 2022-06-22

**Authors:** Gerard Espinosa, Carles Zamora-Martínez, Albert Pérez-Isidro, Daniela Neto, Luz Yadira Bravo-Gallego, Sergio Prieto-González, Odette Viñas, Ana Belen Moreno-Castaño, Estíbaliz Ruiz-Ortiz, Ricard Cervera

**Affiliations:** ^1^ Department of Autoimmune Diseases, Hospital Clínic de Barcelona, Barcelona, Spain; ^2^ Institut d’Investigacions Biomèdiques August Pi i Sunyer (IDIBAPS), University of Barcelona, Barcelona, Spain; ^3^ Department of Internal Medicine, Hospital Clínic de Barcelona, Barcelona, Spain; ^4^ Department of Immunology, Centre de Diagnòstic Biomèdic, Hospital Clínic de Barcelona, Barcelona, Spain; ^5^ Department of Pathology, Center for Biomedical Diagnosis, Hospital Clinic, Barcelona, Spain

**Keywords:** COVID - 19, antiphospholipid antibodies, antiphospholipid syndrome - immunology, diagnosis, thrombosis - immunology, persistence, severe respiratory failure

## Abstract

**Objective:**

Patients with COVID-19 presented with an elevated prevalence of antiphospholipid antibodies (aPL) but the relationship with thrombosis is controversial. We analysed the persistence of aPL and their association with the clinical outcomes during hospitalisation in a cohort of COVID-19 patients.

**Patients and Methods:**

We conducted a prospective study including consecutive hospitalised patients with COVID-19 from Hospital Clínic of Barcelona between March 28th and April 22nd, 2020. Clinical outcomes during hospitalisation were thrombosis, intensive care unit (ICU) admission, and severe ventilatory failure. We determined both criteria and non-criteria aPL. Of note, in those patients with a positive result in the first determination, a second sample separated by at least 12 weeks was drawn to test the persistence of aPL.

**Results:**

One hundred and fifty-eight patients (59.5% men) with a mean age of 61.4 ± 14.9 years old were included. Thrombosis was present in 28 (17.7%) patients, severe respiratory failure in 47 (30.5%), and 30 (18.9%) patients were admitted to ICU. Sixteen (28.6%) patients were positive for the criteria aPL at both determinations and only two (3.6%) of them suffered from thrombosis during hospitalisations (both had aCL IgG). However, they presented with low titers of aCL. Of note, aPL were not related to thrombosis, ICU admission or severe respiratory failure.

**Conclusion:**

Although aPL were prevalent in our cohort of hospitalised COVID-19 patients and they were persistent in half of tested patients, most determinations were at low titers and they were not related to worse clinical outcomes.

## Introduction

The coronavirus disease (COVID-19) caused by the severe acute respiratory syndrome coronavirus 2 (SARS-CoV-2) was first identified in Wuhan, China in December 2019, and rapidly spread globally, leading to a worldwide pandemic (1). Several reports have described a hypercoagulable state, as well as significant changes in haemostatic laboratory parameters (2, 3).

Early reports of COVID-19 already suggested that elevated circulating D-dimer levels might be associated with increased mortality (3). Furthermore and supporting this hypothesis, an autopsy series report demonstrated the presence of fibrin thrombi within distended small vessels and capillaries in the lungs and heart (4).

Since a first small case series report in China (5), several works have described the prevalence of antiphospholipid antibodies (aPL) and their possible association with the development of thrombosis and complications in COVID-19 patients. However, in most studies aPL were measured only at a single timepoint, and information about the methods and quantitative results is scarce. Data from a recent metanalysis revealed that the pooled prevalence rate of one or more aPL - including IgG or IgM isotypes of anticardiolipin (aCL) or anti-β2glycoprotein I (aβ2GPI) or anti-phosphatidylserine/prothrombin antibodies (aPS/PT) or lupus anticoagulant (LAC) - was 46.8% (6). The LAC was the most frequently detected, with pooled prevalence rate of 50.7%, followed by aCL (IgM or IgG) and aβ2GPI (IgM or IgG), with a pooled prevalence rate of 13.9% and 6.7%, respectively (6). Only two studies have retested patients and found a change from positive to negative aPL in almost all the analysed patients suggesting that aPL may be transiently elevated in patients with COVID-19 (7, 8).

Findings are contradictory on the relationship between aPL and hospital outcomes such as mortality, invasive ventilation or venous thrombosis. According to the previous metanalysis, aPL were not significantly associated with the worse clinical outcomes (6).

Given the various gaps in the current understanding of aPL in patients with COVID-19, we aimed to characterise patterns of aPL testing and to describe the clinical course of complications and transient changes of laboratory findings during hospitalisation in a cohort of COVID-19 patients.

## Patients and Methods

### Patients

We conducted a prospective study including consecutive hospitalised patients with COVID-19 from Hospital Clínic of Barcelona between March 28th and April 22nd, 2020. Inclusion criteria were patients aged 18 or older with either suspected (based on clinical, epidemiological and radiological findings) or confirmed (by reverse transcriptase-polymerase chain reaction (RT-PCR) on a nasopharyngeal swab) COVID-19.

Clinical data including demographic variables and comorbidities such as arterial hypertension, diabetes mellitus, obesity, and previous thrombosis were collected. Clinical outcomes during hospitalisation were also recorded. These comprised thrombotic events, intensive care unit (ICU) admission and severe ventilatory failure defined as the ratio of arterial oxygen partial pressure (PaO2 in mmHg) to fractional inspired oxygen (FiO2 expressed as a fraction) ≤ 200 mmHg (9). For thrombosis, the clinical diagnosis was confirmed by objective methods (computed tomography scanning, magnetic resonance imaging, electrocardiographic studies and elevated levels of cardiac enzymes, Doppler ultrasonographic scan, ventilation-perfusion scanning and pulmonary angiography).

This study was conducted in accordance with the principles of the Declaration of Helsinki. Approval was obtained from the Ethical Committee of Hospital Clínic Barcelona (HCB/2020/0727). Written informed consent was obtained from all patients.

### Laboratory Features

#### Biological Biomarkers

The C-reactive protein (CRP), D-dimer, troponin and ferritin levels determined in the first 24 hours of hospital admission were included.

#### aPL Determinations

We determined aPL included in the classification criteria of the antiphospholipid syndrome (APS), i.e., LAC and IgG and IgM isotypes of aCL and aβ2GPI. In addition, aPL which were not included in the classification criteria (the non-criteria aPL) such as IgG and IgM aPS/PT and the IgA isotype of aCL and aB2GPI were also determined. Of note, in those patients with a positive result in the first aPL determination, a second sample separated by at least 12 weeks was drawn to test the persistence of aPL.

#### Lupus Anticoagulant

LAC was detected according to the Clinical and Laboratory Standards Institute (CLSI) 2014 guidelines (10) and to the most recent update of the Subcommittee on Lupus Anticoagulant/Phospholipid-dependent Antibodies of the International Society on Thrombosis and Haemostasis guidelines (11) (**Supplementary Material**).

#### aCL and aβ2GPI Determinations

The study of IgG and IgM aCL and aβ2GPI was performed by chemiluminescence assay (CIA) (QUANTA Flash^®^, Inova Diagnostics, CA). The cut-off recommended by the manufacturer was 20 chemiluminescent units (CU). However, the low/medium cut-off point (the equivalent of < 40 GPL and MPL units) was defined as 95 CU for IgG aCL and 31 for IgM aCL as previously established (12).

#### Non-Criteria aPL Determinations

IgG and IgM aPS/PT determination was performed by enzyme-linked immunosorbent assay (ELISA) (QUANTA Lite^®^, Inova Diagnostics, CA). The cut-off recommended by the manufacturer is 30 U/ml. IgA aCL and aB2GPI were quantified by ELISA (QUANTA Lite^®^, Inova Diagnostics, CA). The cut-off recommended by the manufacturer is 20 U/ml.

### Statystical Analysis

Results of discrete variables were expressed as absolute frequency and percentage. Results of continuous variables were expressed as median accompanied by the 25^th^-75^th^ interquartile range or mean with standard deviation when normally distributed.

Association between qualitative variables was determined with the Exact Fisher test in a contingency table. The relative measure of an effect between subgroups of patients was expressed as odds ratio based on a 95% CI when considering the aPL positivity as the exposure. Mann-Whitney U test was used for comparisons.

Multivariate analyses were performed through the binary logistic regression model using variables that presented a p value <0.05 in a previous univariate analysis and aPL profile. The optimal cut-off points for age, D-dimer, CRP, ferritin and troponin were assessed using the maximum Youden’s Index of the ROC curve analysis for each one of the clinical outcomes. The relative measure of an effect was expressed as odds ratio. Probabilities under 0.05 were considered significant. Data were analysed with IBM SPSS Statistics for Windows, version 23 (IBM Corp., Armonk, N.Y., USA) and GraphPad Prism for Windows, version 8.3.0 (GraphPad Software, La Jolla, California, USA).

## Results

### General Characteristics

Patient population consisted of 94 (59.5%) men and 64 (40.5%) women. Mean age at data collection was 61.4 ± 14.9 years old. Some patients have been included in a previous study (13). Demographic characteristics, comorbidities, laboratory features and outcomes are summarised in **Table 1**. Overall, 8 patients had a known pre-pandemic immunomediated/autoimmune disease. Specifically, two patients had systemic sarcoidosis, two patients psoriasis, and the remaining four patients had membranous glomerulonephritis with anti-PLAR2 antibodies, polymyalgia rheumatica, limited cutaneous systemic sclerosis, and rheumatoid arthritis, respectively. None of them had been tested for aPL pre-pandemic.

**Table 1:** Demographic characteristics, comorbidities, laboratory features, and outcomes of the overall series of patients with COVID-19.

	N (%)
Age (years)	61.4 ± 14.9
Sex (men)	94 (59.5)
Comorbidities	119 (75.3)
Arterial hypertension	69 (43.7)
Dyslipidemia	38 (24.1)
Diabetes mellitus	33 (20.9)
Previous thrombosis	19 (12.0)
Obesity	7 (4.4)
Laboratory features	
C-reactive protein (mg/dL)	5.75 (2.73-10.80)
Ferritin (ng/mL)	627.0 (331.0-1092.5)
D-dimer (ng/mL)	800 (400-1600)
Troponin (ng/L)	9.5 (4.0-24.9)
Outcomes	
Thrombosis during hospital admission	28 (17.7)
Days since symptoms	16.5 ± 7.7
Days since hospital admission	8.2 ± 8.5
Severe respiratory failure ^a^	47 (30.5)
Intensive care unit admission	29 (18.4)
Days since hospital admission	4.5 ± 8.6
Mortality	1 (0.6)
Heparin treatment ^b^	152 (96.2)
Prophylactic dosing	97 (63.8)
Intermediate dosing	26 (17.1)
Anticoagulant dosing	24 (15.8)
Other anticoagulant treatments ^c^	5 (3.3)
Antiphospholipid antibodies	
First sample	158 (100)
Days since symptoms	13.2 ± 8.6
Days since hospital admission	6.2 ± 7.8
Second sample	58 (36.7)
Days between samples	155.9 ± 108.4

Data are presented as mean ± standard deviation, median (interquartilic range), or n (%).

a Data was not available in 4 patients.

b Data was not available in 6 patients.

c Included 3 patients treated with coumadin, one treated with apixaban, and one with fondaparinux.

A positive SARS-CoV-2 RT-PCR was present in 97 (61.4%) patients. Only one patient died due to COVID-19 complications. Considering the main clinical outcomes during hospitalisation, thrombosis was present in 28 (17.7%) patients, severe respiratory failure in 47 (30.5%) and 30 (18.9%) patients were admitted to ICU. Thrombotic events appeared 16.5 ± 7.7 days after the first COVID-19 symptom and 8.2 ± 8.5 days after hospital admission.

### aPL Results

The distribution and prevalence of the classical APS markers (LAC and IgG and IgM aCL and aβ2GPI) as well as non-criteria APS biomarkers (aPS/PT, IgA aCL and aβ2GPI) in the first and second determination are shown in **Table 2**.

**Table 2:** Prevalence and variability of antiphospholipid antibodies in patients with COVID-19.

	First sample (n=158)	Second sample (n=58)	P value
Classification criteria aPL	37 (23.4)	17 (29.3)	0.380
LAC *	24 (21.4)	5 (8.9)	0.052
aCL IgG	11 (7.0)	10 (17.2)	0.036
aCL IgM	5 (3.2)	5 (8.6)	0.137
aβ2GPI IgG	6 (3.8)	4 (6.9)	0.464
aβ2GPI IgM	2 (1.3)	4 (6.9)	0.046
Triple aPL positivity *	1 (0.9)	1 (1.8)	0.466
Non-criteria aPL	30 (19.0)	17 (29.3)	0.136
aCL IgA	2 (1.3)	0	0.605
aβ2GPI IgA	17 (10.8)	10 (17.2)	0.245
aPS/PT IgG	3 (1.9)	1 (1.7)	1
aPS/PT IgM	9 (5.7)	7 (12.1)	0.142
Any aPL	58 (36.7)	28 (48.3)	0.158
Single or multiple aPL profile			
Single aPL			
LAC *	16 (14.3)	2 (3.6)	0.036
Any aCL	6 (3.8) [2]	3 (5.2)	0.704
aCL IgG	3 (1.9)	2 (3.4)	0.612
aCL IgM	2 (1.3) [1]	1 (1.7)	1
aCL IgA	1 (0.6)	0	1
Any aβ2GPI	13 (8.2) [7]	8 (13.8)	0.298
aβ2GPI IgG	0	0	1
aβ2GPI IgM	1 (0.6)	1 (1.7)	0.466
aβ2GPI IgA	12 (7.6) [7]	7 (12.1)	0.292
Any aPS/PT	5 (3.2) [2]	3 (5.2)	0.445
aPS/PT IgG	1 (0.6)	1 (1.7)	0.466
aPS/PT IgM	4 (2.5) [2]	2 (3.4)	0.661
Multiple aPLs			
LAC and aCL *	2 (1.8)	1 (1.8)	1
LAC and aβ2GPI *	3 (2.7)	0	0.552
LAC and aPS/PT *	2 (1.8)	1 (1.8)	1
aCL and aβ2GPI	4 (2.5) [3]	6 (10.3) [1]	0.025
aCL and aPS/PT	1 (0.6)	2 (3.4)	0.176
aβ2GPI and aPS/PT	2 (1.3)	1 (1.7)	1
LAC, aCL and aβ2GPI *	1 (0.9)	0	1
aCL, aβ2GPI and aPS/PT	2 (1.3) [1]	0	1
LAC, aCL, aβ2GPI and aPS/PT *	0	1 (1.8)	0.333

Data are presented as n (%).

Number between [brackets] represent the patient with possible positive LAC result or with LAC test not done.

*N=112 for the first sample and N=56 for the second sample.

aβ2GPI, anti-β2glycoprotein I antibodies; aCL, anticardiolipin antibodies; aPL, antiphospholipid antibodies; aPS/PT, anti-phosphatidylserine/prothrombin antibodies; LAC, lupus anticoagulant.

Overall, 130 out of 158 (82.3%) patients were tested for LAC in the first sample. In 18 patients the LAC result was considered inconclusive and was excluded from the statistical analysis. In 28 patients LAC was not tested. There were no differences in the prevalence of clinical outcomes between the group of patients with positive or negative results of LAC versus those in whom LAC was not tested and those with inconclusive results (data not shown). Patients from the latter group (LAC not tested and with inconclusive results) had higher levels of ferritin (827 ng/mL versus 598 ng/mL; p=0.03). In addition, women were also more numerous in this group (78.3% versus 51.8%; p=0.03).

Considering the first determination, 37 (23.4%) patients were positive for at least one classification criteria aPL and 30 (19.0%) were positive for at least one non-criteria aPL. Twenty-eight out of 158 (17.7%) patients were positive only for classification criteria aPL whereas 21 out of 158 (13.3%) were positive only for non-criteria aPL. LAC was the most frequent aPL at the first determination, present in 24 (21.4%) patients. Of note, we did not find any significant differences in the prevalence of LAC according to the dose of heparin or to the serum level of CRP (data not shown).

We have analyzed the relationship between aPL positivity (any criteria and non-criteria aPL, and any aPL) and previous co-morbidities (dyslipidemia, arterial hypertension, obesity, diabetes mellitus, and previous thrombosis). The analysis has been performed considering the results of first aPL determination and in those patients with persistent aPL. The only significant result was the relationship between the positive result of any aPL in the first determination and previous thrombosis. In fact, 12 (63.2%) patients with any aPL positivity at first determination suffered from previous thrombosis versus 7 (33.8%) who had previous thrombosis but with negative any aPL (p=0.02).

**Figure 1:**
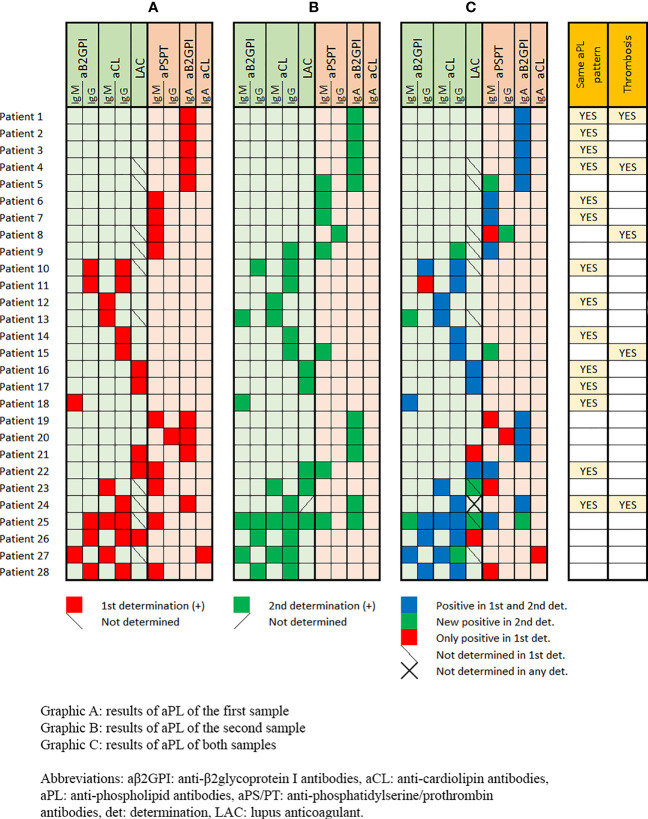
Antiphospholipid antibodies (criteria and non-criteria) profile in the first blood sample (Graphic **A**), in the second blood sample (Graphic **B**), and in both samples (Graphic **C**) of the 28 patients who were positive at both determinations.

Overall, 58 (36.7%) patients were evaluated for both criteria and non-criteria aPL at two temporal points separated by a mean of 155.9 ± 108.4 days. In 12 (20.7%) patients a second sample was collected less than 12 weeks after the first. In two patients, LAC was not tested in the second aPL determination. Overall, 28 out of 56 (50%) patients were positive at both determinations while in the remaining 50% aPL became negative. **Figure 1** describes the aPL (criteria and non-criteria) profile in the first and second blood samples of the 28 patients who were positive at both determinations.

Although 27 out of 56 (48.2%) patients presented with the same aPL (LAC, aCL and/or aβ2GPI) at the two determinations, only 14 (25%) presented with the same aPL profile in both samples. Sixteen (28.6%) patients tested positive for the criteria aPL at both determinations and only two (3.6%) of them suffered from thrombosis during hospitalisations (both had IgG aCL). However, if we consider the cut-off point of 95 CU for IgG aCL CIA assays (the equivalent of < 40 GPL and MPL units measured by ELISA), these two patients presented low levels of IgG aCL (30.8 CU and 26.9 CU in the first determinations and 26.9 CU and 33.6 CU in the second one, respectively). Therefore, no COVID-19 patient in our cohort fulfilled the classification criteria for definite APS. Three additional patients with thrombosis were positive at both aPL determinations, two of them with IgA aβ2GPI as aPL profiles whereas the remainder changed the IgM isotype of aPS/PT of the first sample to the IgG aPS/PT in the second sample (**Figure 1**). One patient was triple positive for the aPL included in the classification criteria in the first sample and one was quadruple aPL positive (LAC plus aCL plus aB2GPI plus aPS/PT) but only in the second sample (**Table 2**). None of the patients suffered from thrombosis during hospitalisation.

Regarding the titers of aPL, 100% of all tests for IgG aCL and 95.8% of those for IgM aCL were below the low/medium cut-off point (≤ 95 CU for aCL IgG and ≤ 31 for aCL IgM). Considering the remaining criteria and non-criteria aPL in patients with positive results, the titers ranged from 22 to 152 CU for IgG aβ2GPI, from 22 to 59 CU for IgM aβ2GPI, from 31 to 91 U/mL for IgG aPS/PT, from 33 to 140 U/mL for IgM aPS/PT, from 24 to 35 U/mL for IgA aCL and from 22 to 127 U/mL for IgA aβ2GPIrespectively.

Of the 16 patients who had positive criteria aPL in the first two determinations, 13 were retested for criteria aPL at follow-up (after a mean of 9.3 ± 3.5 months from the second aPL determination). Most of them remained positive with the same aPL profile (three patients with aCL IgG, two with aCL IgM, two with LAC and one with aβ2GPI IgM). One patient with aCL IgG and another with LAC were negative in the third determination. Finally, three patients had double aPL positivity as their initial aPL profile. One with aCL IgG plus aβ2GPI IgG persisted only with positive aCL IgG, one with aCL IgM plus aβ2GPI IgM persisted only with aCL IgM and the other with aCL IgG and IgM plus aβ2GPI IgG, persisted only with positive aβ2GPI IgG. In all of them, the aPL titers of the third determination were similar to those of the first two. None of these patients suffered from thrombosis or developed autoimmune disease.

### Thrombotic Events During Hospitalisation

Twenty-eight (17.7%) patients presented with thrombosis during hospitalisation. Twenty-seven (96.4%) suffered from pulmonary thromboembolism, three of them with a popliteal deep vein thrombosis, one with a cerebrovascular accident, one with a femoral artery thrombosis and one patient with a subclavian vein thrombosis. An additional patient presented with a cerebrovascular accident.

Patients with thrombosis were older than those without thrombi. Arterial hypertension, obesity and previous thrombosis were present in a higher proportion in the thrombosis group (**Table S1 Supplementary Material**). Regarding the laboratory features at hospital admission, CRP, troponin and D-dimer but not ferritin levels were higher in patients who later developed a thrombotic event during hospitalisation. In our cohort no association was observed between aPL and thrombotic complications. In fact, only two patients were triple and quadruple aPL positive (**Table 2**) but they did not present with thrombosis.

After the univariate analysis for thrombotic events, significant variables (age, arterial hypertension, obesity, previous thrombosis, elevated CRP, troponin and elevated D-dimer) were included in a binary logistic regression analysis with the presence of aPL. Finally, age >60 years, previous thrombosis and elevated D-dimer levels were associated with thrombosis during hospitalisation in our cohort of COVID-19 patients. The odds ratio for each significant variable in the model is shown in **Table 3**.

**Table 3:** Binary logistic regression analysis and odds ratio with 95% confidence interval for clinical outcomes developed during hospitalization.

	*P* value	Odds ratio	95% ConfidenceInterval	
			Lower	Higher
**Thrombotic events**				
Age (>60 years)	0.010	5.63	1.51	21.27
Previous thrombosis	0.137			
Arterial hypertension	0.508			
Obesity	0.009	10.72	1.81	63.43
Elevated D-dimer	0.001	7.52	2.37	23.82
Elevated CRP	0.171			
Any aPL positive	0.824			
Any classification criteria aPL positive	0.149			
Any non-criteria aPL positive	0.863			
**Severe respiratory failure**				
Age (> 55 years)	0.002	5.36	1.89	15.23
Previous thrombosis	0.025	3.59	1.17	11.00
Dyslipidemia	0.264			
Diabetes	0.356			
Elevated D-dimer	0.456			
Elevated CRP	0.349			
Elevated ferritin	0.096			
Any aPL positive	0.792			
Any classification criteria aPL positive	0.611			
Any non-criteria aPL positive	0.510			
**ICU admission**				
Age (> 55 years)	0.010	7.37	1.63	33.38
Sex	0.104			
Previous thrombosis	0.049	3.22	1.01	10.32
Elevated D-dimer	0.456			
Elevated CRP	0.349			
Elevated ferritin	0.096			
aCL IgM	0.077			
Any aPL positive	0.792			
Any classification criteria aPL positive	0.265			
Any non-criteria aPL positive	0.463			

aCL, anticardiolipin antibodies; aPL, antiphospholipid antibodies; CRP, C-reactive protein; ICU, intensive care unit.

### Severe Respiratory Failure During Hospitalisation

Severe respiratory failure was present in 47 (30.5%) patients. As in the case of thrombosis, affected patients were older but no differences were seen related to gender. Diabetes mellitus and dyslipidemia were more frequent in patients with severe respiratory failure. Patients with previous thrombi were more frequent in the severe respiratory failure group (**Table S1 Supplementary Material**). Regarding the laboratory data, ferritin, troponin and D-dimer levels at hospital admission were higher in the severe respiratory failure group. Regarding aPL, no differences were seen between both groups.

After the univariate analysis for severe respiratory failure, significant variables (age, sex, diabetes mellitus, dyslipemia, arterial hypertension, previous thrombosis, elevated ferritin, elevated CRP and elevated D-dimer) were included in a binary logistic regression analysis with aPL. Finally, age >55 years and previous thrombosis were associated with severe respiratory failure in our cohort (**Table 3**).

### Intensive Care Unit Admission During Hospitalisation

Overall, 19 (18.4%) patients needed ICU admission during hospitalisation. In the univariate analysis, patients in the ICU were older and men were more prevalent. Previous thrombosis was present in a higher proportion in the ICU group (**Table S1 Supplementary Material**). Regarding the laboratory data, CRP, ferritin, troponin and D-dimer levels at hospital admission were higher in patients admitted to the ICU. Regarding aPL, IgM aCL were more prevalent in the ICU group.

After the binary logistic regression, including significant variables in the univariate analysis and aPL, age >55 years and previous thrombosis were associated with ICU admission in our cohort (**Table 3**).

## Discussion

In our cohort of hospitalised patients with SARS-CoV-2 infection, the prevalence of aPL determined in only one sample was high, ranging from 19% for non-criteria aPL to 23% for those included in the APS classification criteria. The persistence of aPL, defined as the presence of the same aPL profile, was present in 14 out of 56 (25%) patients who were positive at both determinations. However, no patient fulfilled the classification criteria for definite APS and aPL were not related to worse outcomes of hospitalised patients with COVID-19. Moreover, most positive aPL determinations were at low titers.

Regarding the aPL prevalence in COVID-19 patients, our results are in accordance with previous studies. In the first review of the literature as of June 1, 2020, including 23 studies and 250 COVID-19 patients, 145 (58%) were aPL positive. The most frequent type was LAC, present in 64% of tested COVID-19 patients, followed by aβ2GPI in 13% and aCL in 9% (14). Data from a more recent metanalysis revealed that the pooled prevalence rate of one or more aPL (considering the IgG or IgM isotypes of aCL, aβ2GPI or aPS/PT or LAC) was 46.8%. Specifically, LAC was the most frequent type of aPL, present in 50.7%, followed by aCL in 13.9% and aβ2GPI in 6.7% (6). In a cohort of 172 hospitalised patients with COVID-19, aPS/PT IgG was the most frequent aPL (24%), followed by aCL IgM (23%) and aPS/PT IgM (18%), respectively (15). In this study, LAC was not tested. Finally, in a narrative review, Favarolo et al. (16) found a median incidence of reported cases positive for aPL of 33% (with an interquartilic range of 11% to 52%).

It is important to consider that only a small number of studies has reported the aPL titers. In 31 consecutive COVID-19 patients admitted to the ICU, the titers of IgG aCL ranged from 22.4 to 36.2 U/mL, above the 99th percentile but according to the experience of the authors these titers are “low” positive (17). In this line, Zuo et al. (15) reported a prevalence of aPL of 52% in COVID-19 patients using the manufacturers’ threshold but this decreased to 30% using a more stringent cut-off point (≥ 40 ELISA-specific units). In other study, the prevalence of IgG/IgM aCL and aβ2GPI was tested in 122 critically ill COVID-19 patients and 86 APS patients. Of note, the median levels of IgG/IgM aCL were 15/4 GPL/MPL in COVID-19 patients versus 65/6.2 GPL/MPL in APS patients, respectively. For IgG/IgM aβ2GPI, the levels were 0.06/0.065 optical units in COVID-19 patients versus 1.14/0.23 optical units in APS patients. Cut-off values for IgG/IgM aCL were 20 GPL/MPL and for IgG/IgM aβ2GPI were 0.13 and 0.27 optical units, respectively (18). In other words, titers considered medium or high were described in the minority of COVID-19 patients. In our study, all IgG aCL determinations by CIA in the first and second samples were below 95 UI (the equivalent of 40 GPL and MPL) and the two patients that met APS classification criteria had low levels of IgG aCL (30.8 CU and 26.9 CU in the first determination and 26.9 CU and 33.6 CU in the second), respectively.

We evaluated 58 patients for both criteria and non-criteria aPL at two temporal points separated by a mean of 155.9 ± 108.4 days and the same aPL profile was present in 14 of them (25%). In one study, LAC was repeated in 10 out of 21 critically ill COVID-19 patients that were LAC-positive in the first determination, and 9 of them were LAC negative on the second test performed one month after the first (7). Data from dynamic changes in the titers of aPL in 6 critically ill COVID-19 patients have been described (8). In one patient, medium levels of IgG aβ2GPI persisted after a transient appearance of IgA aβ2GPI plus IgA aCL. In 2 patients, medium levels of IgA aβ2GPI plus IgA aCL persisted after a transient appearance of IgG aβ2GPI. In two additional patients, a transient appearance of aPL was found. Finally, high levels of IgA aCL plus IgA aβ2GPI plus IgG aβ2GPI were found in the remaining patient.

The development of aPL and APS in patients with viral infections is neither new nor exclusive to SARS-CoV-2. Two recent systematic literature reviews described a high prevalence of aPL following different types of viral infections such as human immunodeficiency virus (56%), Epstein-Barr virus (50%), and hepatitis C virus infection (21%) (19, 20). Moreover, 24% of patients fulfilled the classification criteria for APS whereas 44% developed transient aPL with thrombosis and 32% developed transient aPL without thromboembolic events (19).

Thus, the clinical significance of positive aPL tests in COVID-19 patients remains undefined. We found no relationship between aPL and the worse clinical outcomes of patients with COVID-19. Our results are in accordance with those of the metanalysis where there was no association between aPL positivity and mortality, invasive ventilation and venous thromboembolism (6). This apparent lack of correlation between positive aPL and thrombosis raises questions about their pathogenicity in COVID-19 patients. Moreover, published data are also controversial. On the one hand, a group of investigators from Lombardia, Italy, compared the epitope specificity of aβ2GPI found in COVID-19 patients and APS patients (18). Specifically, they assessed the aβ2GPI directed against the N-terminal domain 1 (anti-D1) (anti-aβ2GPI-D1 antibodies are associated with an increased risk of thrombosis and obstetric morbidity in APS patients (21)) or the C-terminal domains 4-5 (anti-aβ2GPI-D4-5) of the molecule. They found that three samples reacted with D1 and three samples tested positive for D4-5. None of the COVID-19 patients with anti-D1 presented with thrombosis. In the group of APS patients, almost all the samples reacted with domain D1 at a high titer (18). On the other hand, Zuo et al. (15) demonstrated the association between high titers of aPL and neutrophil hyperactivity including the release of neutrophil extracellular traps (NETs). Moreover, IgG fractions isolated from COVID-19 patients promoted NETs release from neutrophils isolated from healthy individuals. In addition, injection of IgG purified from COVID-19 patients accelerated venous thrombosis in two mouse models (15). Recently, the same group found that serum samples from COVID-19 hospitalised patients were able to activate endothelial cells compared with those of sepsis patients and healthy controls (22). Interestingly, aCL and aPS/PT from COVID-19 patients strongly correlated with markers of endothelial cell activation and modestly with those of NETs/thrombo-inflammation including C-reactive protein, D-dimer and calprotectin (22).

Our study has some limitations. First, most patients were treated with heparin at the time of LAC testing. Although we tried to minimize the false positive results induced by the heparin interference testing the anti-Xa activity, in 18 (11.4%) patients the results were considered inconclusive and were excluded from the statistical analysis. Second, the quantification of the intrinsic pathway clotting factors could be performed only in some samples because of quantity limitations. Third, LAC was not tested in all patients and in around 20% of them the second sample was collected earlier than 12 weeks after the first. Fourth, a control group of APS patients and healthy individuals to compare the aPL prevalence and their titres was not included. Fifth, the need for ICU admission may be underestimated because the study was carried out at the height of the first wave of the COVID-19 pandemic with a very high number of severe patients admitted and with a limited number of available ICU beds. However, the present work also has remarkable strengths including the determination of both criteria and non-criteria aPL. In addition, we confirmed the aPL presence in a second determination at least 12 weeks apart in most patients who tested positive in the first sample according to the APS classification criteria. Finally, clinical results were robust outcomes with a strict definition.

In the light of our results, prevalence and persistence of both criteria and non-criteria aPL are high in hospitalised COVID-19 patients but most aPL determinations are at low titers and they are not related to the worse clinical outcomes. Further studies are needed to define the exact pathogenic role of aPL in clinical manifestations of COVID-19.

## Data Availability Statement

The raw data supporting the conclusions of this article will be made available by the authors, without undue reservation.

## Ethics Statement

The studies involving human participants were reviewed and approved by Ethical Committee of Hospital Clínic Barcelona (HCB/2020/0727). The patients/participants provided their written informed consent to participate in this study.

## COVAPS-CLINIC Study Group

Members of the COVAPS-CLINIC Study Group. Members are as follows (sorted alphabetically by last name): Alex Almuedo^1^, Giuseppe Barilaro^2^, Luz Yadira Bravo-Gallego^3^, Daniel Camprubí^1^, Júlia Calvo^4^, Aina Capdevila-Reniu^4^, Irene Carbonell^4^, Ricard Cervera^2^, Georgina Espígol-Frigolé^2^, Gerard Espinosa^2^, Cristina Gabara^4^, Priscila Giavedoni^5^, Ignacio Grafia^4^, Andrea Ladino^4^, Gema Maria Lledó-Ibáñez^2^, Ana Matas-García^4^, Pere Millat^1^, Pedro Juan Moreno^4^, Ana Belen Moreno-Castaño^6^, Magdalena Muelas^1^, José Muñoz^1^, José Naval^4^, Joan Padrosa^4^, Martina Pellicé^4^, María Jesús Pinazo^1^, Sergio Prieto-González^4^, Roberto Ríos-Garcés^2^, Natalia Rodríguez^1^, Olga Rodríguez-Núñez^4^, Estibaliz Ruiz-Ortiz^3^, Ruth Sotil^1^, Adrià Tomé^4^, Helena Ventosa^4^, Odette Viñas^3^, and Carles Zamora-Martínez^4^

1. Department of Tropical Medicine and International Health, ISGlobal, Hospital Clínic, Universitat de Barcelona, Barcelona, Catalonia, Spain.

2. Department of Autoimmune Diseases, Hospital Clinic, Institut d’Investigacions Biomèdiques August Pi i Sunyer, University of Barcelona, Barcelona, Catalonia, Spain.

3. Department of Immunology, Hospital Clinic, Institut d’Investigacions Biomèdiques

August Pi i Sunyer, University of Barcelona, Barcelona, Catalonia, Spain.

4. Department of Internal Medicine, Hospital Clinic, Institut d’Investigacions Biomèdiques August Pi i Sunyer, University of Barcelona, Barcelona, Catalonia, Spain.

5. Department of Dermatology, Hospital Clinic, Institut d’Investigacions Biomèdiques

August Pi i Sunyer, University of Barcelona, Barcelona, Catalonia, Spain.

6. Department of Pathology, Center for Biomedical Diagnosis. Hospital Clinic, Institut d’Investigacions Biomèdiques August Pi i Sunyer, University of Barcelona, Barcelona, Catalonia, Catalonia, Spain.

## Author Contributions

The authors as listed on the title page of the manuscript have all made substantial contributions which qualifies them as authors. All authors contributed to critical revisions and approved the final version of the manuscript. GE: design of the study, acquisition of data, analysis, interpretation of data, drafting and revising the article. CZ-M: acquisition of data, drafting and revising the article. AP-I: aPL determinations, analysis, interpretation of data, drafting and revising the article. DN: acquisition of data and revising the article. LB-G: aPL determinations and revising the article. SP-G: design of the study, drafting and revising the article. OV: aPL determinations, analysis, interpretation of data, drafting and revising the article. AM-C: aPL determinations and revising the article. ER-O: aPL determinations, analysis, interpretation of data, drafting and revising the article. RC: design of the study, interpretation of data, drafting and revising the article.

## Conflict of Interest

The authors declare that the research was conducted in the absence of any commercial or financial relationships that could be construed as a potential conflict of interest.

## Publisher’s Note

All claims expressed in this article are solely those of the authors and do not necessarily represent those of their affiliated organizations, or those of the publisher, the editors and the reviewers. Any product that may be evaluated in this article, or claim that may be made by its manufacturer, is not guaranteed or endorsed by the publisher.
