# Supplementary material for: Persistent Antiphospholipid Antibodies Are Not Associated With Worse Clinical Outcomes in a Prospective Cohort of Hospitalised Patients With SARS-CoV-2 Infection
Source: Front Immunol. 2022 Jun 22;13:911979. doi: 10.3389/fimmu.2022.911979 (PMC9257245; doi:10.3389/fimmu.2022.911979)
Supplement: Supplementary file 1 [file Table_1.docx]

Supplementary Material

# Supplementary Table

Table S1: Comparative analysis between hospitalized patients with COVID-19 according to the clinical outcomes during hospitalization.

|  | **Thrombotic**  **events**  **(n=28)** | **No thrombotic events**  **(n=130)** | **P**  **Value** | **Severe**  **respiratory failure**  **(n=47)** | **No severe**  **respiratory failure**  **(n=107)** | **P**  **Value** | **ICU**  **admission**  **(n=29)** | **No ICU**  **admission (n=129)** | **P Value** |
| --- | --- | --- | --- | --- | --- | --- | --- | --- | --- |
| Age (years) | 68.8 ± 10.3 | 59.8 ± 15.3 | 0.004 | 67.0 ± 10.8 | 59.2 ± 15.6 | 0.003 | 66.9 ± 9.7 | 60.2 ± 15.6 | 0.036 |
| Sex (men) | 19 (67.9) | 75 (57.7) | 0.322 | 34 (72.3) | 60 (56.1) | 0.073 | 23 (79.3) | 71 (55.0) | 0.021 |
| Comorbidities  Arterial hypertension  Dyslipidemia  Diabetes mellitus  Previous thrombosis  Obesity | 19 (67.9)  10 (35.7)  7 (25.0)  7 (25.0)  4 (14.3) | 50 (38.5)  28 (21.5)  26 (20.0)  12 (9.2)  3 (2.3) | 0.005  0.113  0.556  0.020  0.005 | 26 (55.3)  18 (38.3)  16 (34.0)  12 (25.5)  2 (4.3) | 42 (39.3)  20 (18.7)  17 (15.9)  7 (6.5)  5 (4.7) | 0.064  0.009  0.011  0.001  0.909 | 16 (55.2)  9 (31.0)  8 (27.6)  8 (27.6)  2 (6.9) | 53 (41.1)  29 (22.5)  25 (19.4)  11 (8.5)  5 (3.9) | 0.214  0.342  0.322  0.009  0.613 |
| Laboratory features  C-reactive protein (mg/dL)  Ferritin (ng/mL)  Troponin (ng/L)  D-dimer (ng/mL) | 11.31 (5.8-20.8)  1196.5 (787.5-1981.5)  19.7 (10.6-40.4)  2150 (1100-9656) | 4.95 (2.5-9.1)  600 (400-1200)  6.7 (3.7-21.0)  600 (400-1200) | 0.001  0.133  0.002  <0.001 | 7.84 (3.3-18.2)  888 (598-1418)  18.0 (7.1-31.1)  1100 (600-3900) | 5.13 (2.7-9.1)  571 (278-880)  6.2 (3.3-14.7)  600 (400-1300) | 0.055  0.001  <0.001  0.005 | 9.3 (4.8-18.9)  888 (598-1702)  27.8 (14.6-40.4)  1400 (600-5500) | 5.2 (2.7-9.4)  608 (305-985)  6.4 (3.6-16.8)  700 (400-1400) | 0.036  0.008  <0.001  0.001 |
| aPL (first sample)  Classification criteria aPL  aCL IgG  aCL IgM  aβ2GPI IgG  aβB2GPI IgM  Lupus Anticoagulant  Triple aPL positivity  Non-criteria aPL  aCL IgA  aβ2GPI IgA  aPS/PT IgG  aPS/PT IgM  Any aPL | 4 (14.3)  3 (10. 7)  0  1 (3.6)  0  2 (10.5)  0  7 (25.0)  1 (3.6)  4 (14.3)  0  1 (3.6)  10 (35.7) | 33 (25.4)  8 (6.2)  5 (3.8)  5 (3.8)  2 (1.5)  22 (23.7)  1 (0.8)  23 (17.7)  1 (0.8)  13 (10.0)  3 (2.3)  8 (6.2)  48 (36.9) | 0.210  0.391  0.293  0.945  0.510  0.206  1  0.373  0.230  0.508  0.419  0.594  0.904 | 10 (21.3)  4 (8.5)  3 (6.4)  3 (6.4)  1 (2.1)  5 (15.6)  0  9 (19.1)  1 (2.1)  5 (10.6)  1 (2.1)  2 (4.3)  17 (36.2) | 23 (21.5)  4 (3.7)  2 (1.9)  1 (0.9)  0  17 (22.4)  0  21 (19.6)  1 (0.9)  12 (11.2)  2 (1.9)  7 (6.5)  37 (34.6) | 0.976  0.219  0.146  0.085  0.130  0.427  1  0.945  0.547  0.916  0.915  0.577  0.849 | 7 (24.1)  4 (13.8)  3 (10.3)  3 (10.3)  1 (3.4)  2 (11.8)  0  6 (20.7)  1 (3.4)  2 (6.9)  0  2 (6.9)  11 (37.9) | 30 (23.3)  7 (5.4)  2 (1.6)  3 (2.3)  1 (0.8)  22 (23.2)  1 (0.8)  24 (18.6)  1 (0.8)  15 (11.6)  2 (2.3)  7 (5.4)  47 (36.4) | 1  0.119  0.043  0.076  0.334  0.520  1  0.796  0.334  0.740  1  0.670  1 |

Data are presented as mean ± standard deviation, median (interquartilic range), or n (%)

Abbreviations: aβ2GPI: anti-β2glycoprotein I antibodies, aCL: anticardiolipin antibodies, aPL: antiphospholipid antibodies, aPS/PT: anti-phosphatidylserine/prothrombin antibodies, ICU: intensive care unit.
