# Supplementary material for: Persistent Antiphospholipid Antibodies Are Not Associated With Worse Clinical Outcomes in a Prospective Cohort of Hospitalised Patients With SARS-CoV-2 Infection
Source: Front Immunol. 2022 Jun 22;13:911979. doi: 10.3389/fimmu.2022.911979 (PMC9257245; doi:10.3389/fimmu.2022.911979)
Supplement: Supplementary file 2 [file DataSheet_1.docx]

Supplementary Material

# Supplementary Data

Determination of lupus anticoagulant (LAC): Blood samples were drawn in 3.2% sodium citrate blood collection tubes. Platelet poor plasma was obtained after centrifugation of citrate blood at 3000 rpm for 15 minutes. Plasma samples were stored frozen at -80ºC. They were thawed at 37ºC and tested within the following 2-4 hours.

LAC was detected according to the Clinical and Laboratory Standards Institute (CLSI) 2014 guidelines (1) and to the last update of the Subcommittee on Lupus Anticoagulant/Phospholipid-dependent Antibodies of the International Society on Thrombosis and Haemostasis guidelines (2). We used a combination of tests for the LAC detection: the diluted Russell viper Venom time test (dRVVT) and an activated partial thromboplastin time (APTT) activated with silica. Both test (LA1 and LA2 for dRVVT, from Siemens, and Cephen LS and Cephen for the silica-APTT test, from Hyphen) were performed in the Atellica Coag360 coagulometer (Siemens Healthineers, Marburg, Germany).

The order of testing of our algorithm implies performing the patient screening time and, if it is found abnormally prolonged (above our local cut-off), proceed with the confirmatory test directly with a reactive that includes an excess of phospholipids. Of note, any elevated screening is not candidate for further considerations if the screening ratio (patients screening time/normal pooled plasma screening time) was <1.20 (Smith 2017).

A normalization of the coagulation time in the confirmation test was indicative of phospholipid dependence and, if the ratio patient screening time/patient confirmation time was >1.20, in any of the two tests, the sample was considered positive. A patient screening/confirm ratio >1.20 without normalization of the confirmation time was followed by a mixing test of the sample with normal pooled plasma, in a 1:1 proportion, to rule out false positive results due to clotting factors deficiencies. Having performed the confirmation assay in the previous step allowed us to detect any phospholipid dependence even when after the mixing test the result is negative due to the dilution effect if the antibody titer is low. These cases were catalogued as a “inconclusive, pending confirmation”.

It is important to highligh that the vast majority of the COVID-19 patients analyzed received heparin at prophylactic, intermediate, or therapeutic doses, depending on the clinical context. In order to minimize the false positive results induced by the heparin interference, the anti-Xa activity was tested in all the patients receiving heparin and with a positive LAC result. The dRVVT reactive includes a heparin neutralizer up to 1IU/mL of anti-Xa activity of heparinemia. On the other hand, our silica-APTT test is extremely sensitive to low doses of heparin above 0.1UI/mL. For this reason, after analyzing the patient anti-Xa activity, if it was below 1UI/mL we assumed that it was neutralized by the dRVVT reagents, and we considered this test a confirmed positive. Nevertheless, an isolated positivity in the APTT test with anti-Xa activities above 0.1 UI/mL was considered a doubtful positive.

The quantification of the intrinsic pathway clotting factors could be performed only in some samples because of quantity limitations. They were analyzed by coagulometric tests, except FVIII quantification that was performed with a chromogenic substract. All clotting factors quantifications were also analyzed in Atellica Coag360 (Siemens Healthineers, Marburg, Germany).

1. Smith LJ, et al. Laboratory diagnosis of the lupus anticoagulant. Clin Lab Sci 2017;30(1):7.
2. Pengo V, Tripodi A, Reber G, Rand JH, Ortel TL, Galli M, et al. Update of the guidelines for lupus anticoagulant detection. J Thromb Haemost 2009;7(10):1737-1740.
